# Application of Dual Metabarcoding Platforms for the Meso- and Macrozooplankton Taxa in the Ross Sea

**DOI:** 10.3390/genes13050922

**Published:** 2022-05-21

**Authors:** Ji-Hyun Lee, Hyoung Sul La, Jeong-Hoon Kim, Wuju Son, Hyun Park, Young-Mog Kim, Hyun-Woo Kim

**Affiliations:** 1 Department of Marine Biology, Pukyong National University, Busan 48513, Korea; jhlee208@pukyong.ac.kr; 2 Division of Ocean Sciences, Korea Polar Research Institute, Incheon 21990, Korea; hsla@kopri.re.kr (H.S.L.); swj5753@kopri.re.kr (W.S.); 3 Department of Polar Sciences, University of Science and Technology, Daejeon 34113, Korea; 4 Division of Life Sciences, Korea Polar Research Institute, Incheon 21990, Korea; jhkim94@kopri.re.kr; 5 Department of Biotechnology, Korea University, Seoul 02841, Korea; hpark@korea.ac.kr; 6 Department of Food Science and Technology, Pukyong National University, Busan 48513, Korea; ymkim@pknu.ac.kr; 7 Marine Integrated Biomedical Technology Center, National Key Research Institutes in Universities, Pukyong National University, Busan 48513, Korea

**Keywords:** Ross Sea, metabarcoding, zooplankton, next-generation sequencing, antarctica

## Abstract

Meso- and macrozooplankton play crucial roles in the trophic web and the biological carbon pump in the ocean by transferring energy from lower to higher trophic levels and vertically exporting carbon from the surface to the deep ocean and seabed. In this study, zooplankton community structures in the Ross Sea, Antarctica, were analyzed using metabarcoding methods. Both regular barcode (RB) (using a PacBio Sequel system) and mini barcode (MB) (using the Illumina MiSeq platform) methods were utilized. As the result of a combination of the two bioinformatic pipelines used in the RB, 55 reliable haplotypes were obtained from the pooled zooplankton net samples, whereas 183 amplicon sequence variants (ASVs) were isolated from the MB metabarcoding analyses of 14 individual stations. Among these, 39 (70.9%) and 125 (90.6%) showed higher than 99% sequence identity to the database, indicating that there were sufficient reference sequences to employ metabarcoding analysis—except for several taxa, including small-sized copepods, cnidarians, and pneumodermatids. A high degree of shared taxa showed that both metabarcoding analyses were feasible for use in the analysis of zooplankton assemblages in the Ross Sea. However, RB would be more useful for the construction of a reference database due to its relatively high cost, whereas MB would be more economic for ecological surveys due to its relatively low cost (albeit, only if reference sequences were well documented using RB). Zooplankton assemblages were highly diverse in each sample site, presumably due to the narrow covered volumes of the vertical net-towed samples from polynyas in the Ross Sea. As metabarcoding data accumulate, we will gain better insights into zooplankton communities and their ecological implications in the Ross Sea.

## 1. Introduction

Climate change has impacted water temperatures and ice conditions, which could alter the base of the food web and subsequent energy flow throughout the entire ecosystem in the Southern Ocean [1,2]. Because zooplankton are easily affected by the physical fluctuations of the oceans that accompany climate change (due to their short lifespan, small body size, and weak swimming capability), they have been used as ecological indicators for global climate change in the ocean [3,4,5,6]. Meso- and macrozooplankton play a crucial role in the trophic web by transferring materials and energy from lower to higher trophic levels in the ocean, as well as in the biological carbon pump by exporting carbon vertically from the surface to the deep ocean and seabed in the ocean [7,8,9]. In addition, they constitute the largest component of zooplankton in terms of biomass and abundance [10]. In particular, several macrozooplankton taxa, including *Euphausia superba*, *Metridia* spp., *Clione* spp., *Limacina helicina*, and Chaetognaths are known to account for almost 90% of the total abundance of zooplankton in the Southern Ocean [11]. For these reasons, many large-scale meso- and macrozooplankton surveys have been conducted to estimate the impact of climate change on the marine ecosystem in the Southern Ocean. For instance, the SCAR Southern Ocean Continuous Plankton Recorder (SO-CPR) survey has documented the zooplankton communities in the Southern Ocean since 1991 [12,13]. However, large-scale surveys require considerable budget and labor [14,15]. Additionally, impacts on the ecosystem during sample collection in a traditional zooplankton survey may not be negligible. For example, the SO-CPR instrument has been towed an estimated distance of 238,000 km and has filtered about a quarter of a trillionth of the total volume of the Southern Ocean, raising the issue of the sustainability of the long-term survey.

Metabarcoding could be a useful alternative to other methods of analysis of zooplankton communities, as it requires lower cost and less labor and has higher sensitivity and accuracy compared with traditional approaches [16]. It has also been useful in identifying new species and cryptic species (species that are difficult to distinguish from one another). Most widely used metabarcoding approaches are conducted based on the comparison of short-read barcodes produced by Illumina or Ion Torrent systems to the reference sequence database [17,18]. Compared with the size of traditional COI barcodes (670 bp), short barcodes of less than 300 bp are often limited in sequence variability to discriminate species. Therefore, a sufficient reference database should be essential for the success of short-read metabarcoding analyses. Alternatively, metabarcoding analysis with long-read sequencing technologies, such as the Pacific Biosciences platform (PacBio, Menlo Park, CA, USA), have been introduced [19,20]. Despite its lower throughput and higher error rates compared with the Illumina system, the PacBio platform could produce accurate data through rigorous quality filtering during the bioinformatic process, suggesting its reliability in metabarcoding analysis. Because regular-sized barcodes can be obtained using this platform, metabarcoding with the PacBio system would be more efficient in species identification. However, its cost would be higher than that accrued obtaining short barcodes; the choice to utilize the metabarcoding platform should be made after considering the purpose of the research.

The reference sequence database for Antarctic organisms has been relatively well established. The Census of Antarctic Marine Life (CAML) program has revealed the unexpected richness of biodiversity in Antarctica through numerous major research voyages. As a result, approximately 14,000 species and 3000 DNA barcodes have been archived in the Register of Antarctic Marine Species (RAMS, http://www.marinespecies.org/rams/index.php, accessed on 23 April 2022) and the Barcode of Life data system (BOLD, https://www.boldsystems.org/, accessed on 23 April 2022), respectively. Notably, DNA barcodes for meroplankton have been well documented in the Ross Sea [21,22]. The Ross Sea is the most productive region in the Southern Ocean, supporting 38% and 25% of the world’s Adelie and Emperor penguins, respectively [23,24]. The substantial reference sequence data in the Ross Sea could help researchers employ metabarcoding for zooplankton surveys with relatively low costs and labor expenditures. Considering the limited number of taxonomic experts and difficulty in accessing the Antarctic Ocean, zooplankton surveys using metabarcoding analysis would be a good alternative to laborious traditional methods. However, to the best of our knowledge, no publication about zooplankton metabarcoding in the Ross Sea has been reported. The main object of the present study was to estimate the current status of reference data for both short and long metabarcoding analyses of zooplankton samples in the Ross Sea and evaluate their feasibility and reliability for further plankton surveys in the Antarctic Ocean. First, we collected plankton net samples from 14 sites in the Ross Sea over 2 years and compared metabarcoding results between the regular barcodes (RBs) with PacBio and mini barcodes (MBs) with an Illumina MiSeq platform. These included taxon coverage and specificity, feasibility for the current reference sequence database, optimization of the bioinformatic pipeline, and qualitative analyses.

## 2. Materials and Methods

### 2.1. Sample Collection and DNA Extraction

**Figure 1:**
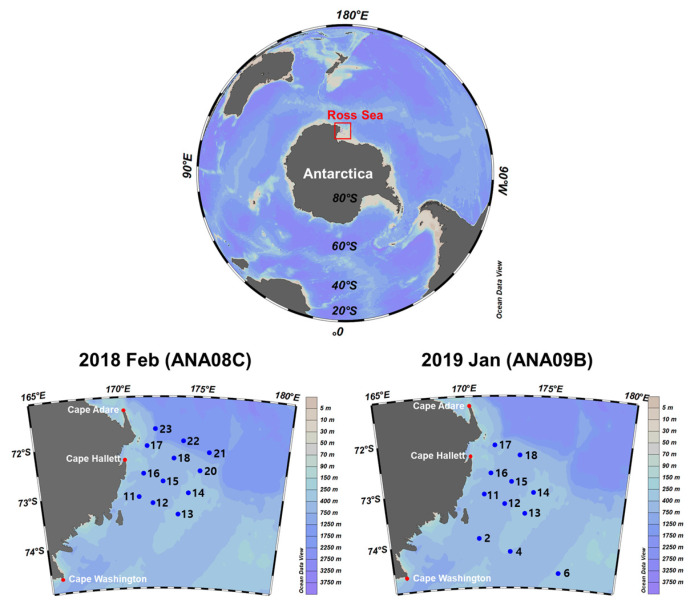
Map of the sampling stations during two IBRV Araon expeditions in the Ross Sea, ANA08C from 26 February to 1 March in 2018 and ANA09B from 16 to 21 January in 2019. Blue dots represent each sampling site in the Ross Sea. Map view created by Ocean Data View software v.5.1.7 (ODV, https://odv.awi.de/, accessed on 23 December 2021).

Plankton surveys were conducted within the Ross Sea as a part of the project ‘Ecosystem Structure and Function of Marine Protected Area (MPA) in Antarctica’ (Figure 1). The survey area was located from 71°41′53″–74°34′16″ to 171°04′23″–176°06′16″ in the Ross Sea. Zooplankton were collected during two expeditions by the Korean icebreaker RV Araon, ANA08C (26 February–1 March in 2018) and ANA09B (16–21 January in 2019). The Bongo net (330 μM mesh size and 0.28 m^2^ mouth opening) was towed vertically from 200 m to the surface at 1.0 m/s during recovery and 0.5 m/s during descent. The net samples were split into 1/2 aliquots using a Folsom splitter [25], poured into a 1 L bottle, and frozen immediately at −80 °C until DNA extraction. After measuring wet weight, each sample was homogenized with 6 volumes of lysis buffer at 3000 rpm for 1 min three times on ice using a WiseTis HG-15D homogenizer (Daihan Scientific Co., Seoul, Korea). Genomic DNA was extracted from homogenized samples using an AccuPrep^®^ genomic DNA extraction kit (Bioneer, Daejeon, Korea) following the manufacturer’s instructions. Isolated genomic DNA was quantified and qualified by an ND-1000 NanoDrop spectrophotometer (Thermo Scientific, Wilmington, DE, USA).

### 2.2. PacBio Amplicon Sequencing for Regular Barcodes (RBs)

In order to prepare the amplicon libraries for single molecular real-time (SMRT) sequencing, two-step PCR was performed according to the PacBio Barcoded Universal Primers for Multiplexing Amplicons protocol (https://www.pacb.com/wp-content/uploads/Procedure-Checklist-Preparing-SMRTbell-Libraries-using-PacBio-Barcoded-Universal-Primers-for-Multiplexing-Amplicons.pdf, accessed on 29 April 2020). Genomic DNA from each station was amplified using a pair of COI universal primer [26] tailed with forward and reverse universal sequences (jgLCO1490: 5′-/5AmMC6/GCAGTCGAACATGTAGCTGACTCAGGTCACTITCIACIAAYCAYAARGAYATTGG-3′ and jgHCO2198: 5′-/5AmMC6/TGGATCACTTGTGCAAGCATCACATCGTAGTAIACYTCIGGRTGICCRAARAAYCA-3′).

PCR amplification was first performed using the following cycling conditions: initial denaturation at 94 °C for 5 min, followed by 35 cycles of 94 °C for 30 s, 48 °C or 30 s, and 72 °C for 30 s, with a final extension of 72 °C for 5 min. The PCR mixture (20 μL) contained 2 μL of template, 1 μL of each primer (10 pmol each), 0.5 μL of dNTPs (10 mM each), 2 μL 10× Ex Taq buffer, 0.2 μL of Ex Taq Hot Start Version (Takara Bio Inc., Tokyo, Japan), and DNase/RNase-free deionized water. The amplified PCR products were separated by electrophoresis 1.5% agarose gel. Amplicons with the expected sizes (approximately 770 bp for zooplankton) were cut and pooled together by year of expedition (ANA08C and ANA09B) and then purified using an AccuPrep^®^ gel purification kit (Bioneer, Daejeon, Korea). The purified products (10 ng) were indexed by a second PCR using 2 μL Pacbio barcoded universal primer (Pacific Bioscience, Menlo Park, CA, USA), 0.5 μL dNTPs (10 mM each), 4 μL 5× Phusion HF buffer, 0.2 μL of Phusion high-fidelity DNA polymerase (1 U) (Takara Bio Inc., Tokyo, Japan), and DNase/RNase-free deionized water in a 20 μL reaction volume. The PCR cycles comprised 98 °C for 1 min, 20 cycles of 98 °C for 15 s, 62 °C for 15 s, 72 °C for 45 s, and, finally, 72 °C for 7 min. The libraries were constructed using a SMRTbell^®^ express template prep kit (Pacific Bioscience, Menlo Park, CA, USA) for the PacBio Sequel platform (Pacific Bioscience, Menlo Park, CA, USA). The quality and quantity of each library were checked using a 2100 bioanalyzer (Agilent Technologies, Palo Alto, CA, USA), and sequencing was performed on a PacBio Sequel system using an SMRT Cell 1M v3.

### 2.3. Illumina Miseq Sequencing for Mini Barcodes (MBs)

To construct libraries for paired-end Illumina sequencing on a MiSeq platform, the extracted genomic DNA samples from 14 different stations (6 for 2018; 8 for 2019) were used as the template for PCR amplification (Figure 1). First, PCR amplicons were prepared through two PCR amplification steps per library using universal COI primers (COIMISQF1 and COIMISQR1) and adapter-linked COI primers (NXCOIMISQF2 and NXCOIMISQF2), following an optimized protocol described previously [27]. Second, libraries were constructed from the purified amplicons as templates using the Nextera XT index kit (Illumina, San Diego, CA, USA). The amplified size was 630 bp containing the primer sequences. The constructed libraries were quantified using a Qubit dsDNA HS assay kit (Invitrogen, USA) and a Quantus fluorometer (Promega, Madison, WI, USA). The quality of each library was checked using a 2100 bioanalyzer (Agilent Technologies, Palo Alto, CA, USA) and pooled in equimolar concentrations. The library was sequenced on an Illumina MiSeq platform using the MiSeq reagent kit v3 (600-cycle) (Illumina, San Diego, CA, USA).

### 2.4. Bioinformatic Analysis of Regular Barcodes (RBs)

**Figure 2:**
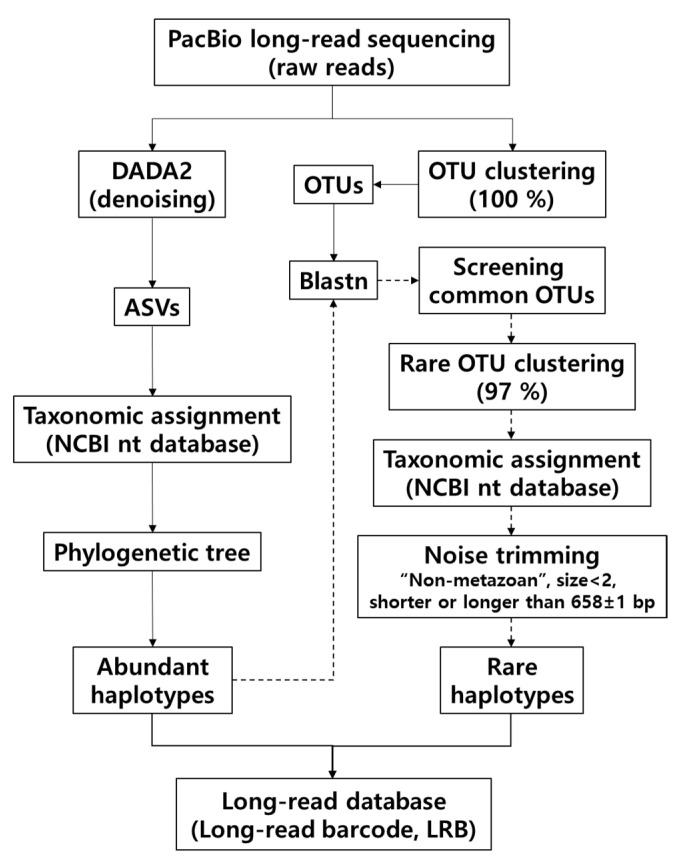
Flow chart of a bioinformatics pipeline for regular barcodes (RBs). The regular-read database was constructed by combining two bioinformatics approaches: denoising and clustering.

Barcoded raw reads were separated by demultiplexing barcodes using SMRT Link software (v6.0.0). To determine the haplotype sequences, the demultiplexed reads were analyzed via two bioinformatics approaches for denoising and clustering, as shown in Figure 2. First, the denoising method was used to obtain abundant haplotypes and remove noisy data. The demultiplexed reads were quality-filtered and denoised using the DADA2 package [28] in R version 4.0.0 [29], according to the online protocol (https://benjjneb.github.io/LRASManuscript/LRASms_Zymo.html, accessed on 21 May 2019). Chimeras were removed using the isBimeraDenovo function. Then, amplicon sequence variants (ASVs) were subjected to local BLAST v.2.9.0 searches against the NCBI nucleotide (nt) database, which was downloaded in December 2020. The ASVs were assigned to the top-hit species or genus with more than 99% or 95% sequence identity in the database, respectively. The ASVs with between 90% and 95% identity were assigned to the family level [30]. The ASVs with less than 90% identity or 50% query coverage were classified as unknown. Then, each haplotype was assigned based on the local BLAST search and phylogenetic tree analysis.

Second, in order to identify rare or low-frequency species, quality filtering and operational taxonomic unit (OTU) clustering of the demultiplexed sequences were performed at 100% similarity using a combination of MOTHUR and UCHIME. Screening of the sequences for expected size and removal of primer sequences were performed using the MOTHUR v.1.41.3 software package [31]. Sequence clustering into OTUs and elimination of chimeric sequences were carried out with UCHIME v.8.1 software [32]. The OTUs were compared for sequence identity to abundant haplotypes obtained using the denoising method. Then, the OTUs with less than 95% sequence identity were reclustered into OTUs at a 97% similarity level. Taxonomic assignment of each OTU was performed using a local BLASTN search (v.2.9.0) against the NCBI-NT database. The OTUs that fit specific criteria (size less than 2 (2 < OTUs), non-metazoan, and shorter or longer than the sequence length of 658 ± 1bp) were removed from further analysis. A haplotype database was constructed by integrating the haplotypes obtained by the two methods and analyzing the phylogenetic tree. Haplotypes with more than 99% sequence identity were assigned to the species name, and those with between 99% and 95% or between 95% and 90% identity were assigned as genus or family names, respectively; the others (<90% identity) were classified as unknown.

### 2.5. Bioinformatic Analysis of Mini Barcodes (MBs)

After paired-end sequencing, adapter and index sequences from raw reads were trimmed, and the reads with low quality (QV < 20) that were less than 100 bases in length were removed from further data analysis using CLC Genomics Workbench v. 8.0 (CLC Bio, Cambridge, MA, USA). The primer sequences were removed with Cutadapt v2.6 [33]. The reads were then processed with the DADA2 package [28] in R version 4.0.0 [29] to determine amplicon sequence variants (ASVs). Initial quality filtering was performed using the filterAndTrim function with the following parameters: maxEE = 2, rm.phix = TRUE, and truncLen = c (270,210). ASVs from the forward and reverse reads were merged with a minimum overlap of 10 bp using the mergePairs function. Chimeric sequences were removed using the BimeraDenovo function. Finally, an ASV count table and sequence file were generated from the 14 sequencing runs. In order to eliminate potential erroneous ASVs, the ASV table was further curated with LULU v.0.1.0 [34]. The taxonomic assignment of each ASV was conducted by the local BLASTN (v.2.9.0) against the NCBI-NT database. Each ASV with sequence identity of more than 98% or 95% was assigned to the top-hit species or taxon, respectively [30]. The ASVs with between 90% and 95% identity were assigned to the family level, and those with less than 90% identity or 50% query coverage were described as unknown. The unknown ASVs were further classified at the phylum level based on phylogenetic tree analysis and BLASTx best hits against the NCBI nonredundant (NR) protein database. Non-metazoan ASVs were eliminated from further analysis. A phylogenetic tree was constructed by the haplotypes (463 bp) obtained from the MB and RB using the maximum likelihood method in Molecular Evolutionary Genetics Analysis X software (v.10.0.5) (Mega-X, Pennsylvania, PA, USA) [35].

After LULU curation and taxonomic classification, the ASV count data were normalized, accounting for the variations in sequencing depth using the estimateSizeFactors function in the DESeq2 package (v.1.32.0) (https://bioconductor.org/packages/release/bioc/html/DESeq2.html, accessed on 5 December 2014) [36]. The proportions of each species or taxon were calculated from the normalized ASV counts. A non-metric multidimensional scaling (NMDS) plot was generated from the normalized count data by the Bray–Curtis similarity matrix using PRIMER v.7 software (https://www.primer-e.com/, accessed on 1 January 2015) [37] to visualize differences between communities. α diversity was calculated from the ASV count data using the estimate_richness function in the phyloseq v.1.36.0 package (https://www.bioconductor.org/packages/release/bioc/html/phyloseq.html, accessed on 22 April 2013) [38].

## 3. Results

### 3.1. Extraction of Haplotypes from Regular Barcodes (RBs) and Mini Barcodes (MBs)

**Figure 3:**
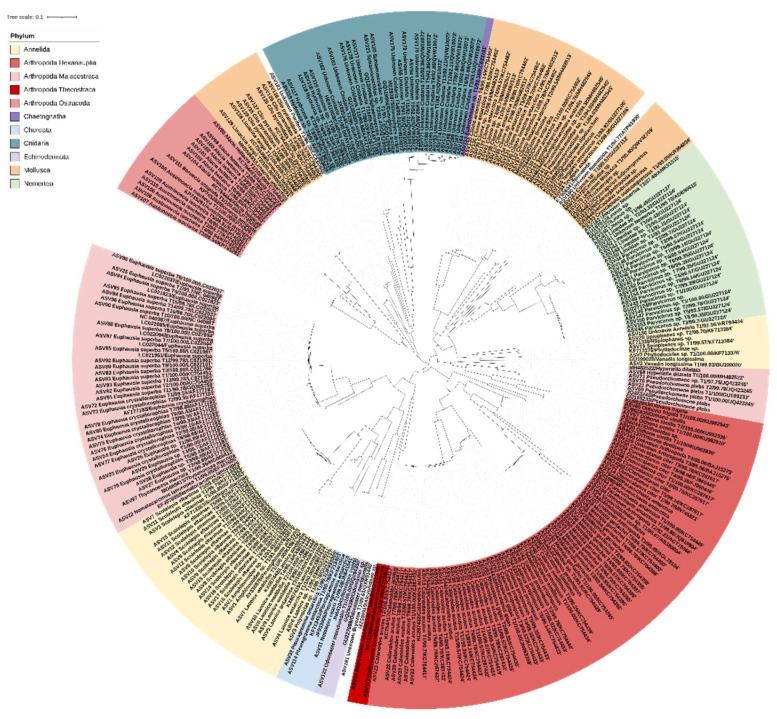
Maximum-likelihood (ML) phylogenetic tree of zooplankton haplotypes identified in the Ross Sea. The tree was constructed by Molecular Evolutionary Genetics Analysis (Mega) X software (v.10.0.5) with 1000 bootstrapping replications. In total, 55 and 183 haplotypes from RB and MB, respectively, and 75 reference sequences from the GenBank database were used for the analysis.

A total of 42,552 and 34,075 raw reads were generated from the regular-read barcoding (RB) results (ANA08C for 2018 and ANA09B for 2019), respectively (Table 1). After filtering and denoising by the DADA2 pipeline, a total of 14,341 (33.7%) and 11,923 (35.0%) reads were obtained, which consisted of 26 and 45 amplicon sequence variants (ASVs), respectively. The 26 and 45 ASVs were inferred from 13,932 and 9881 unique sequences in 2018 and 2019, respectively. Among them, 18 (14,278 reads) and 32 metazoan ASVs (10,798 reads) were identified, respectively. Excluding four common ASVs, 46 ASVs were finally obtained from the RBs of two years of samples (Table 1). Aside from 46 ASVs by the DADA2 pipeline, nine rare OTUs were additionally obtained by the designed OTU clustering pipeline using MOTHUR and UCHIME software (Figure 2). None of those rare OTUs, including *Calanoides acutus*, *Paraeuchaeta antarctica*, *Oithona similis*, *Tergipes antarcticus*, and *Nematocarcinus lanceopes*, were identified among denoised ASVs, indicating the feasibility of the currently designed OTU clustering pipeline (Table 1). A total of 55 putative haplotypes (46 ASVs + 9 OTUs) were ultimately obtained in the RB, consisting of five phyla, including Annelida, Arthropoda, Chordata, Mollusca, and Nemertea (Figure 3). Those five phyla were further classified into seven classes (Polychaeta, Hexanauplia, Thecostraca, Malacostraca, Actinopterygii, Gastropoda, and Pilidiophora), covering 15 orders, 20 families, 20 genera, and 18 species, respectively (Table S1). Species names were assigned for 39 putative haplotypes with 99% and higher similarity to the reference database, whereas the genus names were assigned for 13 putative haplotypes with lower than 99% similarity (Table S1). Three putative haplotypes with low identity (<90%) were assigned as unknown Cnidaria due to the high sequence identity to the hydrozoan, *Dimophyes arctica* (QVW10072) based on the BLASTx and phylogenetic tree analyses. Collectively, the taxonomy for all 55 putative haplotypes obtained by the RB was determined. In 2018, 12 genera and 4 phyla were identified, whereas 15 genera and 5 phyla were identified in 2019 (Table S1). Seven genera were commonly identified in both surveys, including *Scolelepis eltaninae*, *C. acutus*, *Metridia gerlachei*, *Clione*, and *Parvicirrus* sp. The proportions of the commonly identified species were 99.0% and 63.3% in 2018 and 2019, respectively, which indicated that they were among the main zooplankton taxa present in the Ross Sea. A total of 14 haplotypes (36.6%) were exclusively identified in 2019, of which 7 were genus *Euphausia* haplotypes (28.1%), whereas 5 were identified in 2018 only (0.7%), including *Ctenocalanus citer*, *Paraeuchaeta antartica*, and *Notolepis coatsi*.

As a result of the mini-read barcoding (MB), 2,640,586 and 788,096 raw reads were generated from the 14 libraries (6 in ANA08C and 8 in ANA09B, respectively) (Table 1). As a result of bioinformatic processes, including qualify filtering, merging, and chimera removal, a total of 1066 and 721 ASVs were identified from 1,268,522 (48.0%) and 360,336 reads (45.7%), respectively. After screening the artifacts using LULU curation, 206 and 122 ASVs were ultimately obtained (Table 1). Additionally, 60 non-metazoan ASVs were excluded, and 154 and 103 metazoan ASVs were used for further analysis. Excluding 74 commonly identified ASVs, 183 ASVs were considered reliable putative haplotypes in the MB. They were classified into 26 species, 32 genera, 27 families, 17 orders, 11 classes, and 8 phyla (Table 1 and Table S2). Although higher genus numbers were identified in 2018 (31 genera) than in 2019 (18 genera), proportions of 17 commonly identified genera were considerably high, accounting for 83.62% in 2018 and 84.65% in 2019 (Table 2). The most commonly identified species was *M. gerlachei* (30.53% in 2018 and 16.50% in 2019), followed *by Clione limacina antarctica* (30.16% in 2018 and 12.65% in 2019) and *C. acutus* (2.66% in 2018 and 15.15% in 2019). Additionally, 14 genera were exclusively identified in 2018 (4.19%), whereas one species was identified only in 2019 (*P. antarctica,* 0.74%).

A total of 17 genera were commonly identified by both analyses; they accounted for 99.28% of RB and 82.14% of MB, indicating a high degree of taxon coverage of both primer sets (Table 3). Three RB-specific genera, including *Nematocarcinus*, *Notolepis*, and *Cryocapulus*, accounted for only 0.53% of total reads. Compared with those in RB, an 8.3-fold higher proportion of MB-specific genera was identified, accounting for 4.37% of total MB reads. Specifically, eight arthropod genera were exclusively identified in MB, including *Calanus*, *Rhincalanus*, *Hyperiella*, and *Alacia* (Table 3).

### 3.2. Annelida

In total, 12 haplotypes in the phylum Annelida were identified by RB, all of which belonged to the class Polychaeta, consisting of three families, including *Amphinomidae*, *Phyllodocidae*, and *Spionidae* (Figure 3 and Table S1). One haplotype in the *Amphinomidae* showed 93.61% sequence identity to *Paramphinome jeffreysii* (AY838875). Only one species, *Paramphinome australis*, is currently known in the genus, but it was assigned as *Amphinomidae* sp. due to its low sequence identity. One haplotype in the *Phyllodocidae* was assigned as *Vanadis longissima* (GU199020) with 99.03% identity to the database (Table S1). All ten haplotypes in the family *Spionidae* exhibited 99.5% or higher sequence identity to *S. eltaninae* (KF713383)*,* a sole species in the genus, assigning its name.

As in the RB, all three families were also identified by MB (Table 2 and Table S2). Each haplotype in the families *Amphinomidae* and *Phyllodocidae* showed 93.48% and 100% identity to *Paramphinome* sp. (AY838875) and *Phyllodocidae* sp. (KF713376), respectively. Eight haplotypes were identified in the family *Spionidae*, all of which exhibited 98.5% or higher sequence identity to *S. eltaninae*, as shown in RB (Table S2). Additionally, two genera, *Laonice* and *Spionphanes*, were identified by MB. Another two species were identified in the genus *Laonice*: *Laonice antarctica* (KX867435) and *Laonice weddellia* (KX867442). Nucleotide sequence identity between the two sequences was 79.91%, and further study should be conducted. The other haplotype showed 98.7% and 99.5% identity to *Spiophanes* sp. (KF713384). Proportions of two additional genera in MB, *Laonice* and *Spiophanes*, were low, ranging from 0% to 0.05% of total reads, occurring only in 2018 (Table 2). Among the identified annelids, *Scolelepis* occupied the highest proportion in both years, ranging from 0.01 to 10.30%, (Table 2). Interestingly, only a single species, *S. eltaninae*, was identified in 2019 with higher than 0.01%, whereas all five genera were detected in 2018.

### 3.3. Arthropoda

The most haplotypes were identified in the phylum Arthropoda, accounting for a total of 18 out of 55 phyla obtained by RB, 9 of which belonged to the classes Hexanauplia and Malacostraca, respectively (Table S1). All the haplotypes in Hexanauplia showed 99% or higher sequence identity to the database assigning each species name. However, only two haplotypes in the class showed 100% identity to the database: *O. similis* (KU982830) and *P. plebs* (GU109233). Haplotypes in the class Hexanauplia were further classified into two orders: Calanoida and Cyclopoida, among which *C. acutus* (KC754417), *P. antarctica* (JQ819804), and *O. similis* (KU982830) were obtained only by OTU clustering as the rare haplotypes (Table S1). The single haplotype in the class Thecostraca turned out to be *B. corolliforme* (KF713396). In the class Malacostraca, three orders—Amphipoda, Decapoda, and Euphausiacea—were identified (Table S1). One rare haplotype in the RB, *N. lanceopes*, was the only decapod species identified in this study (Table S1). Whereas only one species was obtained in the Amphipoda (*P. plebs*), seven Euphausiid haplotypes were detected, three of which showed a high sequence identity to the reference database (>99%), presenting two well-known species in the Ross Sea, *E. superba* and *E. crystallorophias*. The other four haplotypes (*Euphausia* sp. T1 to T4) showed the highest sequence identity (>98%) to *E. crystallorophias* (AF177183), suggesting that all of them belonged to *E. crystallorophias* (Figure 3 and Table S1).

As a result of the MB analysis, 92 haplotypes were obtained, covering all classes obtained by RB (Table S2). In total, 28 haplotypes representing 9 species were identified by MB, especially those in Ostracoda (14 haplotypes), which included *A. hettacra*, *A. isocheira,* and *B. antipoda* (Table S2). Aside from those in the Ostracoda, the genera *Calanus, Rhincalanus, Scolecitrichidae, Hyperiella*, and *Thysanoessa* were detected by MB (Table 3 and Table S2). Although four haplotypes with low sequence identity (<90%) were assigned as unknown arthropods, their proportion was negligible (<0.03%).

### 3.4. Mollusca

A total of 10 molluscan haplotypes were identified by RB, all of which were Gastropoda (Table S1). Among them, eight belonged to holoplanktonic gastropod families, including *Cliidae*, *Clionidae*, and *Pneumodermatidae*, whereas the other two were either epibiont (*Capulidae*) or demersal (*Tergipedidae*). Among holoplanktonic gastropods, one haplotype in *Cliidae* showed 99.39% identity to *C. pyramidata* (KC754465). Among six haplotypes in *Clionidae*, two showed 99.70% and 99.54% sequence identity, assigned as *C. limacina antarctica* (two haplotypes; MH482513), respectively, whereas the other four (*Clione* sp. T1 to T4) showed low sequence identity to the reference (98.78 to 98.94%). The last holoplanktonic gastropod in the family *Pneumodermatidae* showed 94.83% identity to *Spongiobranchaea australis* (MH482545). According to Life on Earth (https://eol.org, accessed on 23 April 2022), among two species in the genus *Spongiobranchaea*, only *S. australis* was reported in the Southern Ocean; further study should be conducted for accurate identification. Among two non-holoplanktonic gastropod haplotypes, one showed 99.69% identity with *Cryocapulus subcompressus* (KR364834), a small epibiont gastropod on the calcareous tubes of *Serpula narconensis*. The other showed 99.09% identity to *T. antarcticus* (GU227106) which is an autochthonous species to Antarctic sea ice [39]. This haplotype was identified by the low-abundant haplotype pipeline (Figure 2).

A total of 20 molluscan haplotypes were identified by the MB pipeline, covering six families: *Conidae*, *Tergipedidae*, *Cliidae*, *Clionidae*, *Limacinidae*, and *Pneumodermatidae* (Table S2). Although most genera were identified in both analyses, one and two were exclusively identified by RB (*Capulidae*) MB (*Conus* and *Limacina*), respectively. (Table 3). Among 20 molluscan haplotypes, two haplotypes in the *Limacinidae* and one in the *Clionidae* showed 100% sequence identity, whereas those in the *Pneumodermatidae* showed low identity, ranging from 94.38 to 96.76%, as shown in the RBs (Tables S1 and S2).

### 3.5. Nemertea

All 10 nemertean haplotypes obtained by the RB belonged to one family, *Lineidae*, which included only one genus, *Parvicirrus* (Table S1). Seven of them exhibited higher than 99% sequence identity to *Parvicirrus* sp. (GU227124). In fact, *Parvicirrus dubius* (AJ436940) is the only reference sequence in the genus deposited in the GenBank database. Because the sequence identity of the currently identified haplotypes to *P. dubius* ranged from 81.97 to 82.58%, we failed to assign an accurate species name for them.

Similar to those identified by the RB, 13 haplotypes were identified by MB, all of which failed species assignment (Table S2). Among 13 haplotypes in the family *Lineidae*, ten of showed high sequence identity to *Parvicirrus* sp. (G227124), and two haplotypes showed low identity (91.15% and 90.20%). One haplotype exclusively identified by MB in the Nemertea phylum showed 98.49% identity with *Lineus* sp. (GU227127).

### 3.6. Others

In addition to four main phyla, two haplotypes in the phylum Chordata were identified as *N. coatsi* (JN641041, 99.69%) and *Pleuragramma antarcticum* (JF933905, 99.70%) by RB (Table 3 and Table S1). Additional haplotypes belonging to Chaetognatha, Cnidaria, and Echinodermata were identified only by MB (Table 3 and Table S2). The two MB-specific haplotypes belonging to Chaetognatha and Echinodermata showed 92.18% and 100% sequence identity to *Eukrohnia hamata* (KC633101) and *O. meridionalis* (GU227088), respectively (Table S2). In Cnidaria, 7 haplotypes in the class Hydrozoa and 17 unknown haplotypes were obtained by MB. None of the seven cnidarian haplotypes identified by MB were assigned as unknown due to the lack of a reference database. Those 17 unknown haplotypes identified by MB appeared to also be identified by RB. Three unknown haplotypes in the RB showed 92.42% and 91.92% identity with *D. arctica* (QVW10072), indicating cnidarian taxa. Cnidarian haplotypes identified by MB and RB were clustered together, supporting this idea (Figure 3). This result showed that further study should be conducted to supplement the cnidarian reference sequences in the Ross Sea.

### 3.7. Spatiotemporal Distribution of Zooplankton Species Using Metabarcoding Analysis

Spatiotemporal distribution of zooplankton species was further investigated using metabarcoding analysis (Table 2). The average species richness across all sites was 19.8 in 2018, which was approximately twofold higher than in 2019 (10.1). In total, 17 species, including *M. gerlachei*, *C. limacina antarctica*, and *C. acutus*, were commonly identified in both years, accounting for 83.62% in 2018 and 84.65% in 2019 (Table 2). The highest species numbers (26) were identified at St. 13 in 2018, whereas the lowest (5) were found at St. 2 in 2019 (Table 2).

**Figure 4:**
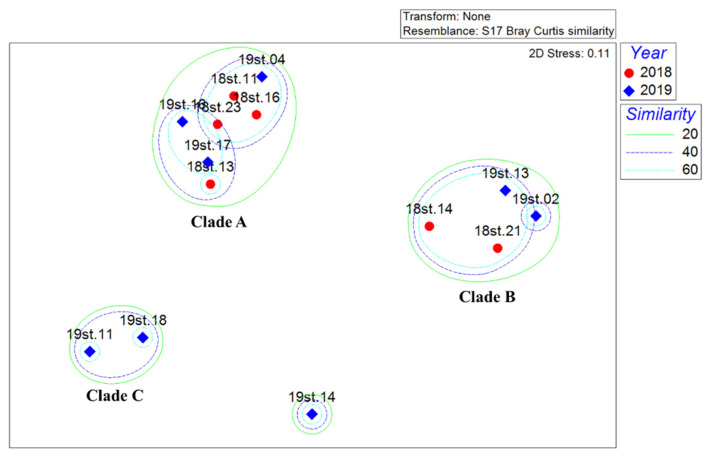
A non-metric multidimensional scaling (NMDS) plot of 14 zooplankton communities based on Bray–Curtis similarity matrix, calculated with the proportions of each species or taxon in the community. Symbols indicate the different sampling years (red circle for 2018; blue diamond for 2019). Green, blue, and light-blue lines represent similarity between the communities of 20%, 40%, and 60%, respectively.

Non-metric multidimensional scaling (NMDS) analysis failed to identify any spatiotemporal implication (Figure 4). Instead, three clades were determined by each main component (*Metridia* for A, *Clione* for B, and *Clio* for C), suggesting each zooplankton sample collected by the vertical plankton net may have contained each plankton patch (Figure 4). Zooplankton composition also differed significantly each year. The most abundant species in 2018 was *M. gerlachei*, with an average proportion of 30.53%, followed by *Clione limacine antarctica* (30.16%) and *A. hettacra* (5.17%), whereas *M. gerlachei* (16.50%) was most abundant in 2019, followed by *C. acutus* (15.15%) and *C. pyramidata* (14.61%). Small-sized copepods, including *Scolecitrichidae* sp. and *O. similis*, as well as one amphipod, *H. dilatata*, were exclusively identified in 2018 (Table 2).

## 4. Discussion

In this study, we applied a metabarcoding strategy for the analysis of zooplankton community structures in the Ross Sea, Antarctica. As a first step, we evaluated the current reference sequence database for both regular-read barcode (RB) obtained by a PacBio Sequel system and the mini-read barcode (MB) generated by the Illumina MiSeq platform. Among 55 haplotypes in the RB and 183 in the MB, 47 (85.45%) and 144 (78.69%) showed higher than 98% sequence identity, respectively, indicating that the reference sequence data for zooplankton taxa in the Ross Sea were relatively good and sufficient for metabarcoding analysis (Tables S1 and S2). Reference sequences in Arthropoda were particularly well documented; 100% in the RB and 92.56% in the MB showed higher than 98% identity, respectively. However, sequences for the small-sized copepods were relatively poorly documented in comparison. Because most zooplankton surveys, including ours, use plankton nets with 330 μm mesh size, mainly due to their versatility for use in the analysis of mesozooplankton (200–2000 μm), the most common size class of the zooplankton community [40]. However, losses of the smaller-sized zooplankton from the nets may have resulted in a lack of their reference sequences in the database, such as those in *Oithonidae*, the most ubiquitous and abundant copepod in the world’s oceans [41,42,43]. Although we found one putative *Oithona* species using RBs, its sequence coverage was only 66%, which suggests that the RB data for those small copepods should be augmented (Table S2). In fact, small-sized or larval copepods generally accounted for 60% of total mesozooplankton abundance cycle strategies of epipelagic copepods in the Southern Ocean [44], and supplements of their reference sequences are required.

Reference sequences for several meroplankton taxa, including pneumodermatids, amphinomids, and lineids, were also limited. Because meroplankton are among the larval or early stages of benthic organisms, the lack of those reference sequences was mainly due to limited information about benthic organisms in the Ross Sea. Zooplankton communities in the Ross Sea could be highly dynamic as a result of various meteorological events, such as polynyas. Metabarcoding of those meroplankton taxa could provide important information to aid in understanding the link between benthic and pelagic communities in the Ross Sea. Additionally, reference sequences for several soft-bodied plankton taxa, including Chaetognatha and Cnidaria, were also limited, presumably due to the difficulty of morphological analysis compared with those with hard shells (Tables S1 and S2). Metabarcoding analysis could aid in understanding those soft-bodied plankton taxa. Collectively, despite limitations in several taxa, we identified that reference sequences for zooplankton in the Ross Sea were relatively well documented. Application of metabarcoding for zooplankton surveys would be possible with supplementation of reference sequences for the several limited taxa identified in this study.

Both universal primers exhibited a high degree of taxon coverage and specificity, among which 99.28% of RB and 82.14% of MB turned out to be shared taxa. Although it appeared that higher numbers of taxa were recovered in MB compared with those in RB, most of the exclusively identified taxa in MB were rare species. The lower cost of MB could lead to researchers producing more read numbers at a similar cost. Considering that numbers were 62-fold higher in 2018 and 23-fold higher in 2019, it would be reasonable to obtain more taxa using MB. Notably, abundant haplotypes in Ostracoda were detected only in the MBs, including *Alacia* spp. and *A. isocheira.* Both genera were widely distributed and abundant in both 2018 and 2019 surveys, up to 12.84% in proportion at St. 23 in 2018 (Table 2). This result appears to be a result of the limited numbers of Ostracoda reference sequences for the RB. The similarity in the COI region of *A. hettacra* (KC754463) to its most closely related species in the database, *Alacia belgicae* (KC754464), was approximately 90%, which suggests that supplementation of the barcodes in Ostracoda in the Ross Sea should be undertaken.

Despite its high cost, there are several merits to adopting RB rather than MB. First, the longer read of the RB contains a higher degree of sequence variations than the MB; several similar taxa can be discriminated only by RB, not MB. For example, we were able to identify two fish species from zooplankton net samples, indicating their ichthyoplankton (eggs or larvae), which could provide useful information about their reproduction ecology. Identification of Antarctic ichthyoplankton using a traditional morphological observation would be challenging. In fact, only a limited number of morphological descriptions for ichthyoplankton have been documented among 322 currently known fish species in the Southern Ocean [45,46]. Some species in the *Nototheniidae* also showed phenotypic plasticity, making accurate identification even more difficult [47]. Therefore, ichthyoplankton monitoring using metabarcoding analysis could be one of the fastest and most reliable methods to provide ecologically important data on the various fish resources inhabiting the Ross Sea. This could prove critical for their scientific management and conservation. One main difficulty is the high degree of DNA sequence similarity within *Nototheniidae* due to the relatively recent evolution of the species in the Southern Ocean [48,49]. Therefore, MB reads may not provide sequence variability enough to discriminate the sister species. RB would be a good alternative, providing the sequence difference required for accurate species identification. Additionally, we were able to identify a high degree of haplotype variations in many taxa using RB (Table S1). Among 55 haplotypes, only two sequences showed 100% identity to the reference database. For instance, six and ten haplotypes were identified in *E. crystallorophias* and *S. eltaninae*, respectively, some of which showed up to 2% sequence differences. Increased regional reference haplotype data would help researchers to expand their knowledge about the zooplankton communities in the Ross Sea beyond species composition, etc. Therefore, metabarcoding analysis using RB would be the most straightforward method to understand the various ecological events that occur in the Ross Sea with low cost and labor requirements.

Despite its merits, the costs of RB using the PacBio Sequel system are still much higher than those for MB using the MiSeq platform. Therefore, the main objective for the construction of the RB could be archiving reliable authentic reference sequences for the zooplankton taxa in the Ross Sea rather than for ecological analysis. Once the long-read reference sequences are sufficient to cover most zooplankton taxa, metabarcoding analysis using MiSeq would be more economic and efficient for ecological studies in the Ross Sea. Therefore, it would be essential to establish a good RB database containing authentic haplotypes for most zooplankton taxa inhabiting the Ross Sea. In order to maximize the authentic haplotype yields with a limited budget, we employed a homemade combination of bioinformatic pipelines for the RBs, in which DADA2 was used to obtain the abundant haplotypes, followed by the extraction of rare haplotypes by filtering out those shared with DADA2 from those generated by the traditional clustering pipeline using MOTHUR and UCHIME (Figure 2). DADA2 is known to be effective in extracting authentic haplotypes, reducing artifacts, such as chimeras, and errors during PCR and sequencing (the main cause of inflated haplotype richness) [28]. However, DADA2 may not be effective at extracting haplotypes with low abundance, which were successfully extracted among those generated by OTU clustering after excluding the OTUs shared with the abundant haplotypes by DADA2 (>95%) (Figure 2). All nine of the low-abundance haplotypes extracted by this bioinformatic pipeline turned out to be unique haplotypes, which were also identified by MB, supporting the reliability of the pipeline we constructed (Tables S1 and S2). Currently, no single bioinformatics pipeline has been endorsed as an authentic method to present species diversity [50], and the currently designed blended methods of two bioinformatic pipelines turned out to be an efficient way to obtain authentic haplotypes from the relatively expensive RB using the PacBio Sequel system. Once authentic reference haplotype sequences are well constructed, researchers could use MBs for zooplankton surveys using metabarcoding, with relatively low cost and high accuracy.

We identified 32 meso- and macrozooplankton genera from 14 stations (6 in 2018 and 8 in 2019,) in the Ross Sea using MB, which was much less than expected, considering the species archive in the Register of Antarctic Marine Species (RAMS; http://www.marinespecies.org/rams/index.php, accessed on 23 April 2022). For example, as a result of expeditions by the Italian National Antarctic Program (PNRA) to the Ross Sea sector from 1987 to 1995, at least 52 genera of copepods were reported [51]. In this study, 10 species, including *M. gerlachei* and *Paraeuchaeta Antarctica*, were reported, most of which were considered dominant. Previous morphological studies have shown that those copepods were the most dominant taxa in the Ross Sea, especially calanoids, and the 32 total genera identified in the MB encompassed most of the zooplankton taxa identified in those studies [52,53]. Ten phyla and more familial taxa were identified, especially in Annelida, Echinodermata, and Nemertea, in DNA-based meroplankton studies using a 100 μm mesh net [21,22]. However, no dominant arthropods were detected—or only one species, *B. corolliforme*, was found. The relatively low species numbers in this study compared to the RAMS database could be explained by the vertical-tow net, which covered only 57 m^3^ of seawater in each station. In fact, the average species numbers obtained by the morphological analysis using the same samples were 4.86, ranging from 2 to 7 at each sample station. We also failed to observe any spatiotemporal clustering in NMDS analysis, presumably due to the sampling method (Figure 4). Alternatively, plankton samples could be collected using a bongo net, which collects more individual samples. Environmental DNA (eDNA) is another method by which to analyze the spatiotemporal distribution of zooplankton taxa in the Ross Sea with relatively low cost and labor requirements. Herein, we collectively employed a metabarcoding technique to elucidate the zooplankton community structure in the Ross Sea. We also evaluated the strong and weak points of two different metabarcoding strategies, RB and MB, and suggested potential applications for each in zooplankton surveys in the Ross Sea. If reference sequences in several taxa were augmented, metabarcoding analysis would become one of the most powerful and feasible methods for zooplankton studies in the Ross Sea.

## 5. Conclusions

Zooplankton community structures in the Ross Sea were analyzed over two years using two metabarcoding methods, RB and MB, utilizing the PacBio Sequel and Illumina MiSeq platforms, respectively. The combination of bioinformatics pipelines for the RBs, denoising and clustering, used in this study led to the successful construction of authentic reference sequences, including abundant and rare haplotypes. Once a sufficient RB database is established that encompasses most zooplankton taxa (especially small copepods and meroplankton), metabarcoding analysis using MiSeq would provide information that could help us to understand shifts or events in zooplankton communities in the Ross Sea ecosystem. Further study is required to supplement the reference sequence database for biodiversity and ecological zooplankton studies in the Ross Sea.

## Figures and Tables

**Table 1:** Summary of two metabarcoding reads.

	Regular Barcoding (RB)				Minibarcoding (MB)	
	Denoised ASVs (Abundant)		Clustered OTUs (Rare)		Denoised ASVs	
	2018	2019	2018	2019	2018	2019
PacBio CCS reads	42,552	34,075	42,552	34,075	n/a	n/a
MiSeq reads	n/a	n/a	n/a	n/a	2,640,586	788,096
Denoised reads	14,341 (33.7%)	11,923 (35.0%)	n/a	n/a	1,268,522 (48.0%)	360,336 (45.7%)
Clustered reads	n/a	n/a	41,119 (96.6%)	33,013 (96.9%)	n/a	n/a
Amplicon sequence variants (ASVs)	26	45	n/a	n/a	206	122
Operational taxonomic units (OTUs)	n/a	n/a	37,778	26,069	n/a	n/a
Metazoan ASVs (reads)	18 (14,278)	32 (10,798)			154 (1,151,931)	103 (232,332)
Non-metazoan ASVs (reads)	8 (63)	13 (1125)			52 (116,591)	19 (128,004)
Putative haplotypes (reads)	46 ASVs (25,076)		9 OTUs (240)		183 ASVs (1,384,263)	
Number of phyla	5				8	
Number of genera	20				32	

**Table 2:** Relative proportions of identified species by mini-read barcoding (MB) in 2018 and 2019.

Phylum	Class	Order	Family	Description	February 2018							January 2019								
					st. 11	st. 13	st. 14	st. 16	st. 21	st. 23	Avg.	st. 2	st. 4	st. 11	st. 13	st. 14	st. 16	st. 17	st. 18	Avg.
Annelida	Polychaeta	Amphinomida	*Amphinomidae*	*Amphinomidae* sp.	0.22	2.58	0.03	2.28		0.00	0.85				0.00			0.00		0.00
		Phyllodocida	*Phyllodocidae*	*Phyllodocidae* sp.		1.31					0.22									0.00
			*Polynoidae*	*Polynoidae* sp.	0.01						0.00									0.00
		Spionida	*Spionidae*	*L. antarctica*	0.00		0.04				0.01									0.00
				*Laonice* sp.					0.02		0.00									0.00
				*Laonice weddellia*	0.05	0.01	0.03	0.00	0.00		0.01									0.00
				*S. eltaninae*	0.42	0.33	0.56	10.30			1.93		4.59	0.01	5.34		0.02	0.01		1.25
				*Spiophanes* sp.		0.01		0.05			0.01									0.00
Arthropoda	Hexanauplia	Calanoida	*Calanidae*	*C. acutus*	3.02	3.35	2.93	3.36	1.73	1.56	2.66		1.79	0.02	0.01	96.37	1.11	16.78	5.11	15.15
				*Calanus propinquus*	1.02	0.33	0.01	0.26	0.02	0.58	0.37		0.38	0.01			0.60	1.69	9.43	1.51
				*Calanus simillimus*	0.05	0.02		0.00	0.00	0.00	0.01							0.11	0.52	0.08
				*Calanidae* sp.					0.01		0.00									0.00
			*Clausocalanidae*	*C. citer*	14.55	6.24	6.52	0.00	0.01	0.33	4.61		0.51							0.06
			*Euchaetidae*	*P. antarctica*	0.13	22.60	0.18	0.75	0.01	1.32	4.16		0.26	0.00		0.25	0.19	0.01		0.09
			*Metridinidae*	*M. gerlachei*	64.51	7.40	4.20	53.03	0.03	54.01	30.53		70.28	0.03	0.01	2.09	32.38	19.12	8.13	16.50
			*Rhincalanidae*	*Rhincalanus gigas*						0.09	0.02							0.12		0.01
			*Scolecitrichidae*	*Scolecitrichidae* sp.	0.01						0.00									0.00
		Cyclopoida	*Oithonidae*	*Oithona frigida*	0.01	0.06	0.40		0.01	0.16	0.11			0.02						0.00
				*O. similis*	0.17	0.06	0.01				0.04									0.00
	Thecostraca	Balanomorpha	*Bathylasmatidae*	*Bathylasma corolliforme*	0.00						0.00									0.00
	Malacostraca	Amphipoda	*Hyperiidae*	*Hyperiella dilatata*	8.20						1.37									0.00
			*Tryphosidae*	*Pseudorchomene plebs*		0.12					0.02		1.79	50.31	0.00			0.01		6.51
				*Pseudorchomene* sp.							0.00			0.02						0.00
		Euphausiacea	*Euphausiidae*	*Euphausia crystallorophias*	0.00	0.01	0.02	1.08			0.19	61.27	1.02	0.01	20.90			0.03	0.00	10.41
				*E. superba*	0.47	0.09	1.10	0.13		6.97	1.46	0.08	2.68	0.04	0.25	0.01	0.28	0.55	3.23	0.89
				*Thysanoessa macrura*					1.09		0.18									0.00
	Ostracoda	Halocyprida	*Halocyprididae*	*Alacia hettacra*	2.73	6.12	0.15	9.19	0.00	12.84	5.17		2.42	0.11				0.14		0.33
				*Austrinoecia isocheira*		0.09	0.04	0.00	0.06	1.68	0.31							0.01		0.00
				*Boroecia antipoda*		0.02					0.00									0.00
Chaetognatha	Sagittoidea	Phragmophora	*Eukrohniidae*	*Eukrohniidae* sp.					0.00		0.00									0.00
Chordata	Actinopterygii	Perciformes	*Nototheniidae*	*P. antarctica*							0.00	5.95								0.74
Cnidaria	Hydrozoa			*Hydrozoa* sp.	3.46	0.15				0.44	0.67									0.00
		Siphonophorae	*Sphaeronectidae*	*Sphaeronectidae* sp.	0.02	0.01			0.00	0.23	0.04									0.00
Echinodermata	Asteroidea	Valvatida	*Odontasteridae*	*Odontaster meridionalis*			0.04				0.01									0.00
Mollusca	Gastropoda	Neogastropoda	*Conidae*	*Conus* sp.				0.02			0.00									0.00
		Nudibranchia	*Tergipedidae*	*T. antarcticus*				14.71			2.45									0.00
		Pteropoda	*Cliidae*	*Clio pyramidata*						0.13	0.02		0.64	42.59	0.00	0.00	0.04	0.05	73.58	14.61
			*Clionidae*	*C. limacina antarctica*	0.01	3.26	80.75	0.04	96.87	0.01	30.16	32.19	0.26	0.07	68.67	0.02		0.04	0.00	12.65
			*Limacinidae*	*Limacina rangii*	0.08	0.22	0.27				0.10									0.00
			*Pneumodermatidae*	*Spongiobranchaea* sp.		0.20					0.03			0.03			17.33			2.17
Nemertea	Pilidiophora	Heteronemertea	*Lineidae*	*Lineus* sp.		0.02					0.00									0.00
				*Parvicirrus* sp.	0.82	0.03	0.01	4.52	0.09	0.34	0.97	0.51	13.27	0.12	4.81	0.37		0.13		2.40
Unknown	Unknown	Unknown	Unknown	Unknown_Annelida						0.00	0.00									0.00
				Unknown_Arthropoda	0.04	0.01				0.01	0.01							0.03		0.00
				Unknown_Bryozoa	0.00						0.00									0.00
				Unknown_Cnidaria	0.00	45.34	2.72	0.29	0.04	19.01	11.23		0.13	6.36	0.00	0.90	48.06	60.01	0.00	14.43
				Unknown_Mollusca		0.01			0.00	0.26	0.05									0.00
				Unknown_Nematoda							0.00			0.24						0.03
				Unknown_Nemertea		0.01					0.00							1.15		0.14
				Unknown_Porifera						0.01	0.00									0.00
Number of Genus					18	21	16	16	14	15	16.7	4	12	13	9	7	8	14	6	9.1
Number of Species					24	26	19	18	15	17	19.8	5	13	14	10	7	8	16	8	10.1

**Table 3:** Comparison of genera or families obtained by two metabarcoding analyses.

	Platform	Shared Genera or Families	Regular-Read Bacodes (RB)	Mini-Read Barcodes (MB)
Phylum				
Annelida		*Amphinomidae* *Vanadis*		*Polynoidae* (0%) *Laonice* (0%) *Spiophanes* (0.01%)
Arthropoda		* *Calanoides* Ctenocalanus * *Paraeuchaeta* *Metridia* Bathylasma * *Oithona* *Pseudorchomene* *Euphausia*	* *Nematocarcinus* (0%)	*Calanus* (1.07%) *Rhincalanus* (0.02%) *Scolecitrichidae* (0%) *Hyperiella* (0.59%) *Thysanoessa* (0.08%) *Alacia* (2.41%) *Austrinoecia* (0.13%) *Boroecia* (0%)
Chaetognatha				*Eukrohniidae* (0%)
Chordata		*Pleuragramma*	*Notolepis* (0.31%)	
Cnidaria				*Sphaeronectes* (0.02%)
Echinodermata				*Odontaster* (0%)
Mollusca		* *Tergipes* *Clio* *Clione* *Pneumodermatidae*	*Cryocapulus* (0.22%)	*Conus* (0%) *Limacina* (0.04%)
Nemertea		*Parvicirrus*		*Lineus* (0%)
Total genera		17	3	17

* rare haplotypes obtained by RB.

## Data Availability

The sequencing data in this study are registered with the NCBI database under BioProject ID PRJNA825464.

## Supplementary Materials

The following supporting information can be downloaded at: https://www.mdpi.com/article/10.3390/genes13050922/s1, Table S1: Meso- and macrozooplankton haplotypes obtained from the regular barcode (RB) by two bioinformatics approaches, denoising and clustering, using the Pacbio platform; Table S2: Meso- and macrozooplankton haplotypes obtained from the mini barcode (MB) using the MiSeq platform.
